# Effects of environmental impact labels on the sustainability of food purchases: A randomised controlled trial in an experimental online supermarket

**DOI:** 10.1371/journal.pone.0309386

**Published:** 2024-09-03

**Authors:** Christina Potter, Rachel Pechey, Michael Clark, Kerstin Frie, Paul A. Bateman, Brian Cook, Cristina Stewart, Carmen Piernas, John Lynch, Mike Rayner, Joseph Poore, Susan A. Jebb

**Affiliations:** 1 Nuffield Department of Primary Care Health Sciences, Radcliffe Observatory Quarter, University of Oxford, Oxford, United Kingdom; 2 Oxford Martin Programme on the Future of Food and Nuffield Department of Population Health, University of Oxford, Oxford, United Kingdom; 3 Department of Zoology, University of Oxford, Oxford, United Kingdom; 4 School of Geography & Environment, University of Oxford, Oxford, United Kingdom; 5 Smith School of Enterprise and Environment, University of Oxford, Oxford, United Kingdom; 6 Department of Physics, University of Oxford, Clarendon Laboratory, Oxford, United Kingdom; PLoS ONE, UNITED STATES OF AMERICA

## Abstract

Providing consumers with product-specific environmental impact information for food products (ecolabels) may promote more sustainable purchasing, needed to meet global environmental targets. This UK study (N = 1051 participants) investigated the effectiveness of different ecolabels using an experimental online supermarket platform, comparing three labels against control (no label). Significant reductions were found in the environmental impact score (EIS) for all labels compared to control (labels presented: values for four environmental indicators [-3.9 percentiles, 95%CIs: -5.3, -2.6]; a composite score [taking values from A to E; -3.9, 95%CIs: -5.2,-2.5]; or both together [-3.2, 95%CIs: -4.5, -1.9]). Providing ecolabels is a promising intervention to promote the selection of more sustainable products.

## Introduction

There is an urgent need to move towards more sustainable diets to mitigate climate change, biodiversity loss, water pollution, unsustainable water use, and other harmful impacts of the current food system on the natural environment [1]. The environmental impacts of different types of foods are highly variable, with up to a 200-fold difference in impact between protein-rich foods (such as beef versus tofu), and 50-fold difference between the same product offered by different producers [2]. However, a lack of product-specific environmental information means that consumers have no easy way to differentiate between more and less sustainable products.

For consumers to be able to make environmentally informed purchases, they need relevant information about the environmental impacts of individual food products at point of choice. Health-related nutrition labelling on foods is now widely implemented, with research showing such labels lead to changes in consumer purchasing and consumption behaviours, for example by reducing purchasing of energy-dense food and drinks and increasing purchasing of items with claimed health-related benefits [3]. Accordingly, one potentially promising approach to encourage more environmentally-sustainable food selection is through the use of environmental impact labels [4].

So-called ‘ecolabels’ typically consist of claims, warnings, or information provided with a product advising consumers about the quality, features or production methods that reduce or increase environmental impacts [5]. One limitation to many of the ecolabels is that they do not capture the environmental impacts from the full lifecycle of food production. Instead, they primarily indicate whether one set of production practices (for example, organic agriculture) or set of standards (for example, Rainforest Alliance) has been applied. However, adoption of certain practices or standards does not necessarily lead to low environmental impacts across multiple indicators [2]. There are increasing societal demands for greater transparency in reporting food production methods that will enable greater precision and accuracy in quantitative ecolabelling [6].

According to the Ecolabel Index, the largest global directory of ecolabels, there are at least 121 ecolabels related to food worldwide [7]. There is great diversity in the type of information conveyed and the contexts within which ecolabels are presented, both of which may impact effectiveness [8]. For example, there are variations in ecolabel formats (e.g. logo vs text only) and the specificity of the information provided (numerical score vs grade vs claim only).

A systematic review of 76 ecolabelling interventions found that ecolabels, across a broad range of formats and content types, are effective at promoting the selection, purchase, and consumption of food and drink products [4]. However, the lack of standardized ecolabel formatting and the diversity of labels may create confusion rather than clarity for consumers [9]. In addition, different consumer groups may respond to ecolabels in different ways [10]. Moreover, while studies have shown ecolabels to be effective at altering food purchasing or selection, these have often featured in relatively small numbers of products and/or participants, limiting the ability to examine effectiveness across the basket and different demographic groups [11–13].

This paper describes an experimental proof-of-principle study that aimed to assess the potential of different environmental impact label designs to effectively promote the selection of lower environmental impact products. These environmental impact labels reflected the broader environmental impact of products, based on Life Cycle Assessment (LCA) data and four environmental impact indicators [14], rather than information on specific production practices and standards (as often the case with many current ecolabels).

The paper is a republication of a previous article (https://doi.org/10.1371/journal.pone.0272800) which the authors asked to be retracted, due to an error being discovered in Study 2, whereby a minority of products were shown with the wrong ecolabel. This study has been removed from the current paper.

## Methods

The study protocol was prospectively registered online (ISRCTN Ref. 15655434). It was reviewed by, and received ethics approval through, the University of Oxford Central University Research Ethics Committee [R65010/RE001]. Informed (written) consent was obtained from all participants, who were recruited between 22^nd^ Jan 2020 and 24^th^ Feb 2020.

### Participants

Adult participants aged 18 years or over were recruited from an online research platform (Prolific Academic, https://www.prolific.co). Panel members who self-identified as vegetarian or vegan (groups that comprise approximately 7% of the UK population [15]) were excluded because some of the products on the shopping list included meat and dairy (given the substantial contribution of these products to the environmental impact from diets) and we wanted to ensure that participants felt able to follow this list when instructed to shop for foods they would be willing to eat. Only English-speaking panel members currently residing in the United Kingdom were eligible.

### Study design and interventions

**Fig 1:**
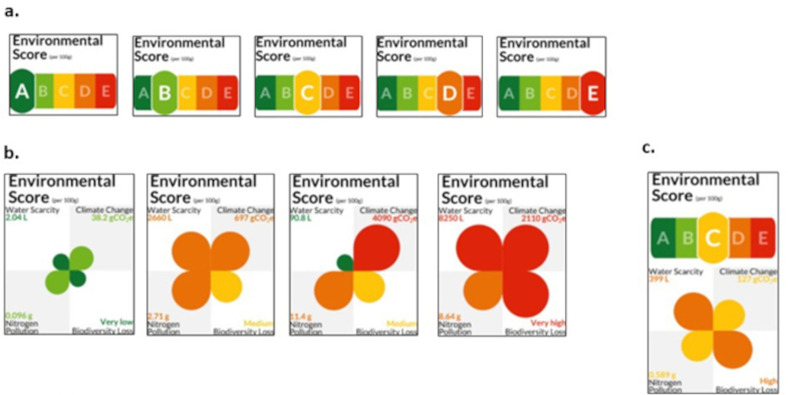
Ecolabels tested. **a**) A-E label: A total composite environmental impact score on an A-E scale with a traffic light colour gradient with five colours ranging from dark green to dark red; **b**) Petal label: Displaying four environmental impact values and using text, colour, and “petal” size as cues; **c**) Combined label: Displaying labels **a** and **b**, above.

In this 2x2 factorial randomised controlled trial (RCT) participants were randomised to one of four study conditions: 3 intervention (different labels) and 1 control (no label). Each intervention condition tested one of three labels: A-E (a total composite environmental impact score on an A-E scale with a traffic light colours); Petal (displaying four environmental impact values and using text, colour, and “petal” size as cues), and Combined (both A-E and Petal) (see **Fig 1**; further label details provided below).

The study was conducted in February 2020, using an experimental online supermarket platform developed at the University of Oxford. The supermarket was designed to emulate a real online supermarket for research purposes, the key difference being that food/drink selections are not paid for or received. In the absence of a real purchasing context in which different types of interventions can be implemented, this offers a relatively naturalistic setting to assess a range of labelling options. The site was populated with approximately 20,000 supermarket products (including their images, prices and nutritional information) drawn from foodDB (April 2019), an up to date database of food and drink products available for purchase in six UK online grocery retailers [16]. Data were collected and managed using the supermarket platform and REDCap (Research Electronic Data Capture) tools hosted at the University of Oxford [17,18]. Participants were electronically randomised (ensuring allocation concealment) using REDCap.

#### Labels

Environmental impact scores for product labels were generated using the ingredient lists available for each product and were reported per 100g of each product. Information on the ingredients list was used to:

1. identify the relative composition of ingredients if available (e.g. 10% ingredient X);
2. estimate the relative composition of ingredients where composition information was not provided, using information from similar products and UK labelling regulations;
3. link each ingredient to a global environmental LCA database; and
4. calculate the environmental impact per 100g of product for four environmental indicators (greenhouse gas emissions, scarcity weighted water stress (hereafter water use), land use related biodiversity loss (hereafter biodiversity loss), and eutrophication potential) based on the composition of each ingredient, the type of ingredient (e.g. a mushroom, a tomato, or poultry meat), and environmental information in the database.

More information on the derivation of the environmental impact scores is described in detail elsewhere [14].

We did not have access to data on producers for individual products. Rather than assuming the same values for each product (e.g. fresh berries), which would mean that all such products would have the same (or a very similar) environmental impact score, we identified individual producers with environmental performance data equivalent to the 25^th^ percentile (e.g. a more sustainable producer), 50^th^ percentile (e.g. the median sustainable producer), and 75^th^ percentile impacts (e.g. a less sustainable producer) across all producers for that food category and for each environmental indicator. When calculating the environmental impact scores (as described above), we then randomly assigned products to have all their ingredients sourced from a more sustainable producer, a median sustainable producer, or a less sustainable producer. This ensured that there was variability in the environmental impact scores between products within a given product category, and also that the environmental impact scores were based on producer-level environmental performance data rather than, for example, assuming that more sustainable products had a 20% lower environmental impact.

The four environmental indicators were then condensed into a product-specific environmental impact score. To do this, products were ranked based on their percentile score (rather than absolute values for each of the four indicators). To arrive at a single environmental impact score for each product, we then took the mean percentile across the four indicators, and then re-ranked this overall environmental score such that it ranged from 1 (lowest impact product) to 100 (highest impact product) based on the percentile environmental impact score of each product. To obtain A-E grades, we then split the environmental impact score into quintiles, whereby a value of A = an environmental impact score of 1–20, B = 21–40, C = 41–60, D = 61–80, E = 81–100. We placed equal weighting on each environmental indicator because our focus was on assessing consumer responses to different labels rather than deeper exploration of the grading scheme itself.

An individualised logo for each product was created using environmental impact data using an automated script written in R (see **Fig 1**). Images (.jpg) of each logo were uploaded onto the virtual online supermarket platform and linked to each individual food product, to be displayed underneath the food product during the experiment.

Labels were developed and refined prior to study launch using insights from focus group sessions with UK adults [19] (see **S1 File** for more information on label development). They were displayed on all products (see **S2 File** in S1 File for a list of food groups).

### Procedure

Following online screening questions to ensure eligibility, participants provided electronic (written) consent. Eligible participants were then directed to the supermarket platform, which participants interact with in the same way as a real online supermarket, but with no money being spent and no items received. In line with previous studies using the experimental supermarket platform, participants were asked to select groceries from a shopping list covering 10 items, with no set budget (see **S3 File** in S1 File for an example of the welcome screen on the shopping platform). The food items included in the list were chosen because of the wide variation in environmental impact between these categories. The items on the shopping list were as follows:

- A savoury snack for right now
- Milk for everyday use
- A ready meal
- Cheese to use in a sandwich or light meal
- A pizza (fresh or frozen)
- A bar of chocolate
- Nuts for snacking on
- Meat, fish, or vegetarian alternative protein for main meal
- Rice to accompany the main meal
- Berries for dessert (fresh or frozen)

After completing the shopping task, participants were redirected to a post-test survey where they provided basic demographic information, as well as details concerning their household size and online grocery shopping habits (see **S4 File** in S1 File). A free-text response option enabled them to describe their experience using the supermarket.

### Outcomes

#### Primary outcome

The primary outcome was the mean environmental impact score (hereafter: EIS) of products placed in the shopping basket. A mean environmental impact score of 1 would mean that only those products with the best environmental impact per 100g (falling into the 1^st^ percentile) were selected, while a score of 100 would mean only those with the worst impact per 100g (falling into the 100^th^ percentile) had been chosen.

#### Secondary outcomes

The study had three secondary outcomes. First, we examined the means across products placed in the basket for four individual environmental indicators that the environmental impact scores were based on: i) greenhouse gas emissions (kg CO_2_e), ii) water use (litres), iii) biodiversity loss (species lost x 10^−14^) and iv) eutrophication potential (gPO_4_^3-^e), each per 100g of product. These analyses were conducted using the absolute values of indicators (rather than percentiles as in the EIS), logged to improve model fit (the indicators were highly skewed, with the majority of products having low scores on each indicator, while a few had particularly high scores). An exploratory secondary outcome examined the total environmental impact of shopping baskets, based on weighting each of the four environmental indicators equally.

Second, we explored differences in the nutritional composition of the shopping basket, as healthier foods may be more sustainable [20], including total energy (kcal), energy density (kcal/100g), salt (g/100g), and total carbohydrate, fibre, fat, saturated fat, and sugar, as % energy. We expressed these nutrients as % total energy to place the focus on the nutritional composition of the foods selected and not the absolute quantity of food purchased.

Finally, we examined differences in the overall spend on the shopping basket, expressed as £/100g.

### Sample size

To determine the sample size, we used standard deviation values from a pilot study with 90 people. The study was powered at 90% to detect an absolute difference of 6% (SD1 = 18.9%, SD2 = 22.5%) in the total EIS between each intervention group and control. Based on our calculation, with a two-sided α =  0.05 and allowing for a 15% non-compliance and attrition rate, we required a sample size of *n* = 283 per group (total *N* = 1,132). The study was not powered to examine differences between intervention groups.

Sample size calculations were based on the pilot study method for calculating environmental impact scores (taking percentiles of scores across the sample)–this was discontinued for the main study, due to concerns relating to this potentially being influenced by sample characteristics or the impacts of included study conditions. The rescaled outcome measures have smaller absolute differences, but correspondingly smaller variance, so are not expected to impact study power (and sensitivity analyses using this alternative show similar results).

### Analysis

The primary aim was to estimate the effect on the EIS when products were presented with environmental impact labels compared to control (no labels).

All participant data were screened by two independent researchers to determine eligibility for inclusion in the analysis. This involved meeting a minimum threshold (to allow calculation of the primary outcome), namely buying product(s) from at least 5 out of 10 categories on the shopping list. This acted both as a quality control check, and helped ensure comparability between participants. Beyond this criterion, an intention-to-treat approach was taken whereby all participants were entered into analyses (e.g. if participants bought more than the 10 requested items). An exploratory sensitivity analysis was conducted on just those participants who purchased 10 items.

We used linear regression to estimate the effectiveness of the labels compared to control. Participant characteristics were included in an adjusted linear regression model, to explore associations between participant characteristics and the EIS (see **S4 File** in S1 File for survey questions and coding). Age and gender were included as factors in the adjusted model. Linear regression was used to explore the effects on individual environmental indicator values, the nutrient composition and spend on the shopping basket between conditions.

To determine whether label effectiveness varied due to participant characteristics, we ran separate linear regression models including interaction terms between each participant characteristic variable and intervention condition in exploratory analyses (using a threshold of p<0.05). Statistical analyses were conducted in STATA (Stata Statistical Software: Release 14. College Station, TX: StataCorp LP). The statistician was blinded to group allocation.

## Results

### Participant characteristics

Participants (*N* = 1,051) were on average 38 years old (SD = 12.7 years), 59.5% were female, and 68.8% had shopped online for groceries in the last year. Participants selected a mean of 10.7 (SD = 4.2; **Table 1**) products. See **S2 File** for CONSORT Flow Diagram.

**Table 1:** Baseline characteristics of participants.

	Control	Petal label	A-E label	Combined labels	Total
N	263	262	265	261	1051
**Age, years, mean + SD**	38.1 + 12.6	37.6 + 12.8	38.5 + 13.0	37.8 + 12.6	38.0 + 12.7
**Age category, n (%)**					
**18–20 years**	17 (6.5)	12 (4.5)	14 (5.3)	14 (5.4)	57 (5.4)
**21–40 years**	144 (54.8)	148 (56.5)	146 (55.1)	149 (57.1)	587 (55.9)
**41–60 years**	94 (35.7)	84 (32.1)	87 (32.8)	80 (30.7)	345 (32.8)
**61+ years**	8 (3.0)	18 (6.9)	18 (6.8)	18 (6.9)	62 (5.9)
**Gender, % female**	60.8	63.7	56.3	57.4	59.5
**Household size, mean + SD**	2.9 + 1.4	2.9 + 1.3	3.1 + 3.3	2.9 + 1.4	2.9 + 2.0
**Items purchased, mean + SD**	10.6 +3.6	10.3 +1.4	11.0 +4.3	11.1 +6.1	10.7 + 4.2
**Online shopping, n (%)**					
**Never or not in last year**	75 (28.5)	92 (35.5)	77 (29.3)	81 (31.4)	325 (31.2)
**1–3 times in last year**	68 (25.9)	66 (25.5)	61 (23.2)	60 (23.3)	255 (24.5)
**4–11 times in last year**	65 (24.7)	52 (20.1)	65 (24.7)	66 (25.6)	248 (23.8)
**1–3 times per month**	30 (11.4)	28 (10.8)	37 (14.1)	37 (14.3)	132 (12.7)
**Once per week or more**	25 (9.5)	21 (8.1)	23 (8.8)	14 (5.4)	83 (8.0)

### Primary outcome: Effects of ecolabels on sustainable purchasing

**Fig 2:**
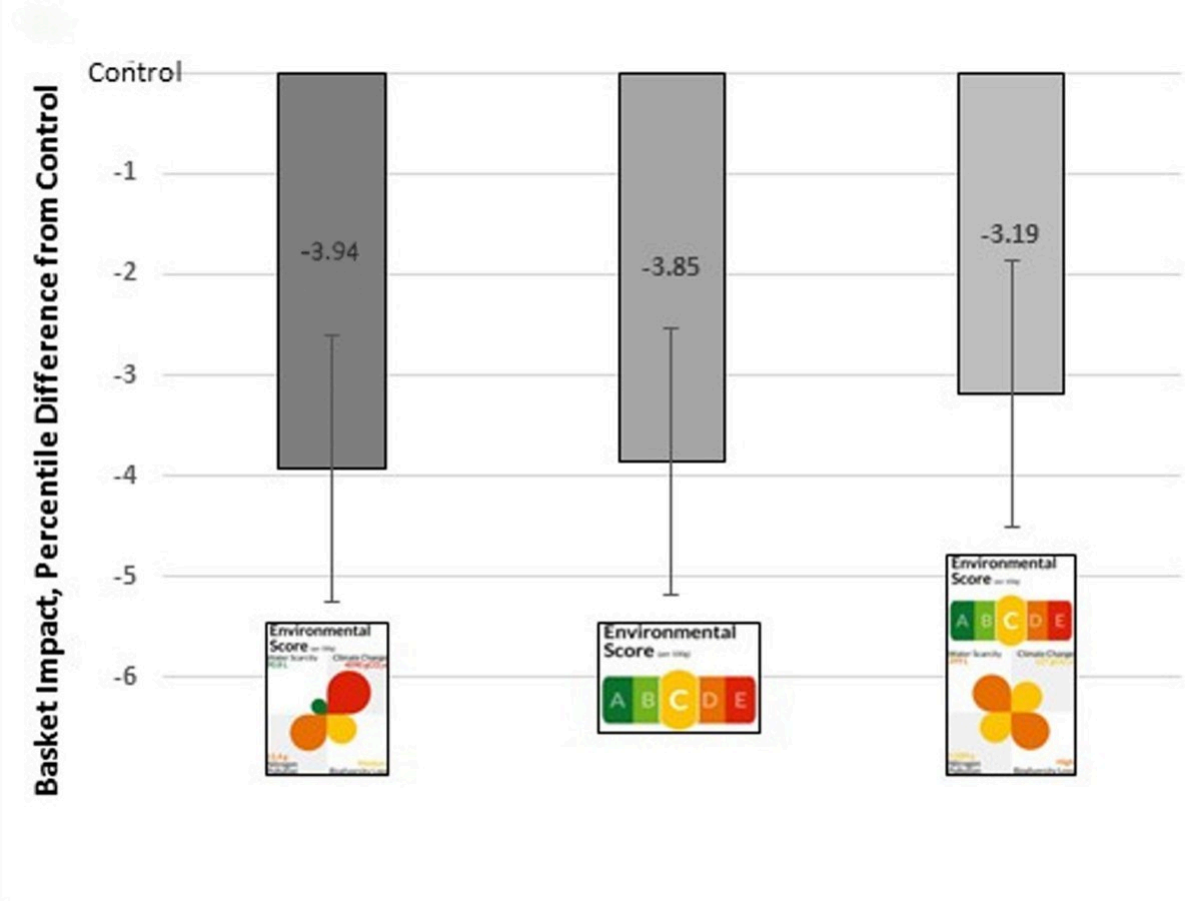
Difference in environmental impact scores. EIS (in percentiles), with 95% CIs, compared to Control (marked as X = 0).

There was a significant reduction in the EIS compared with control (mean EIS = 61.9 (62^nd^ percentile) for all ecolabels: Petal label (mean difference = -3.9 percentiles, 95%CI: -5.3 to -2.6, *p*< 0.001), the A-E label (mean difference = -3.9 percentiles, 95%CI: -5.2 to -2.5, *p*< 0.001), and Combined label (mean difference = -3.2 percentiles, 95%CI: -4.5 to -1.9, *p*< 0.001) (**S1 Table** Model 1, **Fig 2**). Exploratory analyses examining total environmental impact of shopping baskets also found significant reductions for each label (**S2B Table**). Moreover, because the change in the percentile score may not be indicative of the absolute change in environmental impact, the change in impacts for each of the four environmental indicators was also investigated (**Table 2**). The presence of an ecolabel consistently decreased the absolute impact for every environmental indicator: across conditions, there were significant reductions for greenhouse gas emissions (range: -13.9 to -14.8%), biodiversity loss (range: -12.2 to -15.6%), eutrophication potential (-11.3% to -13.9%), and water use (-18.9 to -25.9%).

**Table 2:** Comparison of the individual environmental impact indicators between trial groups.

	Control	Petal vs Control		A-E vs Control		Combined vs Control	
n	263	262		265		261	
	Mean (95%CIs)	Coeff. (95% CI)	% Change	Coeff. (95% CI)	% Change	Coeff. (95% CI)	% Change
**Greenhouse gas emissions**	0.49 (0.4, 0.51)	-0.16 (-0.23, -0.09)**	-14.8%	-0.16 (-0.23, -0.10)**	-14.8%	-0.15 (-0.22, -0.08)**	-13.9%
**Biodiversity loss**	12.1 (11.6, 12.7)	-0.16 (-0.23, -0.08)**	-14.8%	-0.17 (-0.24, -0.09)**	-15.6%	-0.13 (-0.21, -0.05)*	-12.2%
**Eutrophication potential**	2.0 (1.9, 2.1)	-0.15 (-0.22, -0.08)**	-13.9%	-0.15 (-0.22, -0.08)**	-13.9%	-0.12 (-0.19, -0.05)*	-11.3%
**Water use**	2027.9 (1919.8, 2142.)	-0.27 (-0.36, -0.18)**	-23.7%	-0.30 (-0.39, -0.21)**	-25.9%	-0.21 (-0.30, -0.12)**	-18.9%

*Note*. Values are geometric means in column 1 and model coefficients with dependent variables being the natural logs of individual environmental indicators (95% CIs) in the columns 2, 4, and 6. %Change is calculated based on the exponentiated coefficients for individual environmental indicator scores

*p < .05

**p < = 0.001.

A sensitivity analysis examining effects in those who purchased 10 items, as asked on the shopping list, showed similar patterns of results (**S2B Table).**

### Secondary outcomes: Effects of ecolabels on the spend and nutrient composition of the basket

There was a small (£0.02 /100g) but statistically significant increase in the spend on the shopping basket for the Petal label compared to control (**Table 3**). There were no differences between any of the label conditions and control in the total energy, sugar, fibre, protein, or salt content of the shopping basket (**Table 3**). Only the A-E label condition had baskets with lower fat and saturated fat content.

**Table 3:** Comparison of spend and nutrient composition of the shopping basket between trial groups.

	Control	Petal label vs Control	A-E label vs Control	Combined vs Control
n	263	262	265	261
**Spend, £/100 g**	0.52 ± 0.14	0.02 (0.00, 0.05)*	-0.01 (-0.03, 0.01)	0.00 (-0.02, 0.02)
**Energy, kcal/g**	1.90 ± 0.40	0.04 (-0.03, 0.11)	0.01 (-0.05, 0.09)	0.04 (-0.03, 0.10)
**Fat, %energy**	44.2 ± 8.1	-1.4 (-2.8, 0.1)	-1.6 (-3.1, -0.2)*	-0.8 (-2.3, 0.7)
**Saturated fat, %energy**	18.3 ± 4.3	-0.7 (-1.4, 0.1)	-0.8 (-1.5, -0.3)*	-0.4 (-1.1, 0.4)
**Carbohydrate %energy**	34.3 ± 9.8	1.7 (0.0, 3.5)	1.7 (-0.1, 3.5)	1.1 (-0.7, 2.9)
**Sugar, %energy**	10.4 ± 3.8	-0.4 (-1.0, 0.2)	-0.4 (-1.0, 0.3)	-0.2 (-0.9, 0.4)
**Protein, %energy**	19.8 ± 3.9	-0.5 (-1.2, 0.2)	-0.2 (-0.9, 0.5)	-0.3 (-1.0, 0.4)
**Fibre, g/100g**	1.11 ± 0.44	0.04 (-0.03, 0.12)	0.02 (-0.06, 0.09)	0.04 (-0.03, 0.11)
**Salt, g/100g**	0.54 ± 0.86	0.01 (-0.12, 0.13)	-0.07 (-0.20, 0.05)	-0.08 (-0.20, 0.05)

### Exploratory analyses

The adjusted linear regression models showed that the EIS values increased with age (**S1 Table** Model 2). There was no evidence of an effect of gender on the EIS. Interaction effects suggested the Petal and Combined labels may be more effective for younger age groups, whereas there was no evidence of differential impact of A-E labels by age (**S1 Table** Model 4).

## Discussion

Meeting global climate targets will require a rapid reduction in diet-related environmental impacts. This proof-of-principle experiment shows that each of the environmental impact labels tested were effective at encouraging the selection of products with lower environmental impact scores across four environmental indicators (i.e. greenhouse gas emissions, water use, land use related biodiversity loss and eutrophication potential).

### Policy implications

This research supports previous findings suggesting traffic light environmental impact labels were effective at increasing the sustainability of product selections [13]. This study suggests that policies introducing ecolabels could be effective at changing consumer behaviour to increase the sustainability of food purchases. Given more sustainable foods tend to also be healthier [20], it is possible that presenting these alongside nutrition labels–which may already be on packs in some contexts–may often provide complementary purchase cues. However, further exploration of how nutrition and environmental labels could interact is needed–given that presenting one may change consumer perceptions of the other [21], and/or consumers may prioritise health impacts over environmental ones [22], potentially limiting the impact of the latter when discrepancies in label values occur.

This study also provides insights into the types of ecolabels that may be most effective. The Petal label was the only label that displayed values for each environmental indicator separately, thus providing transparency and detailed environmental impact information about a product. This could have advantages in focusing industry change across a broader spectrum of environmental concerns, compared to labels focused on a single indicator–or to a lesser extent a composite whereby a change to one component could change the overall score. While this label was effective, our focus groups reported that this format was confusing. Interaction analyses suggested the Petal label may be more effective for younger adults, who selected lower environmental impact products, and may have higher motivation to seek more detailed information. The Petal label also was the only label to result in the selection of more expensive shopping baskets relative to control, though the effect size was small. Despite these potential limitations, further work is needed to identify ways to encourage both industry and consumer consideration of a range of environmental concerns.

While we focused on changes in consumer demand, environmental impact labels could also prompt transitions throughout food supply chains, for instance by producers competing to reduce the impacts of their production systems, retailers promoting more sustainable products through price promotions, product placement and in-store marketing, or processors reformulating products to avoid higher-impact ingredients. Providing product-specific environmental impact labels could be an important step to help us reach the target of creating food systems that are compatible with global environmental targets.

### Strengths and limitations

The strengths of this study include the RCT design, high completion rate, and blinded statistical analysis. The use of a large number of products (>20,000) that are present in real supermarkets and a bespoke virtual grocery store website encouraged an engaging online shopping experience. Limitations include the experimental nature of the study. Since participants were shopping for hypothetical foods in an experimental supermarket and not exchanging real money or receiving food, there is the risk that they may have selected lower-impact foods because they were aware of the study aims (perhaps particularly given they were recruited through market research panels and may therefore be more aware of study design), though this effect is minimised for comparisons between labels. Indeed, while previous studies examining nutritional labelling using experimental supermarkets have suggested that the effect sizes in experimental studies may be larger than seen in real purchasing contexts, the pattern of results has been found to be relatively consistent between findings in these experiments and real purchases [23,24]. The shopping list comprised a limited number of categories of products, and will not reflect the range of ways in which people shop [25]. Future work could examine differences in selections without the use of a shopping list, and also include additional incentives to maximise the probability that items selected are those participants would purchase, such as informing participants they would receive some of the items selected [23,24]. Moreover, we introduced additional variability in environmental impacts related to different producers of the same product: specifically, we randomly assigned a 25th percentile, 50th percentile, or 75th percentile impact to ingredients of a product. It was important to include such variability as it exists in the real world, but future work will seek to use possible correlates of environmental impact (such as country of origin; whether the food is organically produced; etc.) to introduce this variance to ensure greater accuracy. We also excluded vegetarians and vegans in this study, for whom the relative effectiveness of ecolabels may differ. Given these limitations, the size of effects of the environmental impact labels may differ in a real shopping context where a broader range of products are likely to be purchased and where there may be less variation in label values within certain food categories. However, there is no reason why this should affect the findings regarding the relative effectiveness of different labels.

The focus of this paper was on the label format and not the methodology underlying the label values (considered elsewhere [14]). There are a variety of means to normalise and weight different indicators in life cycle impact assessments, however, and there is ongoing discussion as to their merits [26]. Future work could determine which weighting across indicators might be most effective at promoting sustainable purchasing behaviour, as well as how this weighting might vary across national and regional contexts.

## Conclusion

Providing product-specific environmental impact labels at point of choice during grocery shopping may be a promising intervention to promote the selection of more sustainable food products. There is a plethora of eco-labelling schemes on the market and detailed evaluation of their performance in real-world retail environments is needed to identify the label layouts that most effectively promote sustainable purchasing behaviour. The current study highlights the promise of using a single environmental impact score, collating information from multiple different environmental indicators, which is applied across all products. Rapid development of a single consistent and effective label format is likely to be important for consumer awareness and if these are to play an effective role alongside other measures targeting behaviour change at the population-level.

## Supporting information

S1 File Process of label development; Categories of food groups displayed on the supermarket platform; Woods experimental online supermarket platform welcome screen; Post-test survey. (DOCX)

S2 File CONSORT flow diagram. (DOC)

S1 Table Linear regression models. (PDF)

S2 Table S2A and S2B Tables. Comparison of the mean environmental impact score between trial groups; Exploratory analyses. (DOCX)

S3 Table Percentage (n) of purchased items falling into each environmental impact grade, and the mean (s.d.) score on each environmental indicator of purchased items by grade. (DOCX)

## References

[1] Willett W, Rockström J, Loken B, Springmann M, Lang T, Vermeulen S, et al. Food in the Anthropocene: the EAT–Lancet Commission on healthy diets from sustainable food systems. The Lancet. 2019;393(10170):447–92. doi: 10.1016/S0140-6736(18)31788-4 30660336

[2] Poore J, Nemecek T. Reducing food’s environmental impacts through producers and consumers. Science. 2018;360(6392):987–92. doi: 10.1126/science.aaq0216 29853680

[3] Crockett RA, King SE, Marteau TM, Prevost AT, Bignardi G, Roberts NW, et al. Nutritional labelling for healthier food or non‐alcoholic drink purchasing and consumption. Cochrane Database of Systematic Reviews. 2018:27;2(2):CD009315. doi: 10.1002/14651858.CD009315.pub2 29482264 PMC5846184

[4] Potter C, Bastounis A, Hartmann-Boyce J, Stewart C, Frie K, Bianchi F, et al. The effectiveness of environmental sustainability labels on the selection, purchase, or consumption of food and drink products: a systematic review. Environment and Behavior. 2021; 53(8):891–925. 34456340 10.1177/0013916521995473 PMC8384304

[5] Thøgersen J, Haugaard P, Olesen A. Consumer responses to ecolabels. European Journal of Marketing. 2010;44(11/12):1787–810.

[6] D’Amico P, Armani A, Gianfaldoni D, Guidi A. New provisions for the labelling of fishery and aquaculture products: Difficulties in the implementation of Regulation (EU) n. 1379/2013. Marine Policy. 2016;71:147–56.

[7] Ecolabel Index. Ecolabel Index–Global directory of ecolabels 2019 [Available from: http://www.ecolabelindex.com/.

[8] Ibanez L. Ecolabels: Are they environmental-friendly? Encyclopedia of Law and Economics. 2016:1–9.

[9] Moon SJ, Costello JP, Koo DM. The impact of consumer confusion from eco-labels on negative WOM, distrust, and dissatisfaction. International Journal of Advertising. 2017;36(2):246–71.

[10] Teisl MF, Rubin J, Noblet CL. Non-dirty dancing? Interactions between eco-labels and consumers. Journal of Economic Psychology. 2008;29(2):140–59.

[11] Kanay A, Hilton D, Charalambides L, Corrégé J-B, Inaudi E, Waroquier L, et al. Making the carbon basket count: Goal setting promotes sustainable consumption in a simulated online supermarket. Journal of Economic Psychology. 2021;83:102348.

[12] Vanclay JK, Shortiss J, Aulsebrook S, Gillespie AM, Howell BC, Johanni R, et al. Customer Response to Carbon Labelling of Groceries. Journal of Consumer Policy. 2011;34(1):153–60.

[13] Muller L, Lacroix A, Ruffieux B. Environmental Labelling and Consumption Changes: A Food Choice Experiment. Environmental and Resource Economics. 2019;73(3):871–97.

[14] Clark M, Springmann M, Rayner M, Scarborough P, Hill J, Tilman D, et al. Estimating the environmental impacts of 57,000 food products. PNAS. 2022; 119 (33) e2120584119. doi: 10.1073/pnas.2120584119 35939701 PMC9388151

[15] YouGov. Dietary choices of Brits 2022 [Available from: https://yougov.co.uk/topics/lifestyle/trackers/dietery-choices-of-brits-eg-vegeterian-flexitarian-meat-eater-etc.

[16] Harrington RA, Adhikari V, Rayner M, Scarborough P. Nutrient composition databases in the age of big data: foodDB, a comprehensive, real-time database infrastructure. BMJ Open. 2019;9(6):e026652. doi: 10.1136/bmjopen-2018-026652 31253615 PMC6609072

[17] Harris PA, Taylor R, Minor BL, Elliott V, Fernandez M, O’Neal L, et al. The REDCap consortium: Building an international community of software platform partners. Journal of Biomedical Informatics. 2019;95:103208. doi: 10.1016/j.jbi.2019.103208 31078660 PMC7254481

[18] Harris PA, Taylor R, Thielke R, Payne J, Gonzalez N, Conde JG. Research electronic data capture (REDCap)—A metadata-driven methodology and workflow process for providing translational research informatics support. Journal of Biomedical Informatics. 2009;42(2):377–81. doi: 10.1016/j.jbi.2008.08.010 18929686 PMC2700030

[19] Potter C. LEAP Conducts Focus Groups on Environmental Labelling of Food Products 2019 [Available from: https://www.leap.ox.ac.uk/article/leap-conducts-focus-groups-on-environmental-labelling-of-food-products.

[20] Clark M, Springmann M, Hill J, Tilman D. Multiple health and environmental impacts of foods. Proceedings of the National Academy of Sciences. 2019;116(46):23357–62. doi: 10.1073/pnas.1906908116 31659030 PMC6859310

[21] Jürkenbeck K, Sanchez-Siles L, Siegrist M. Nutri-Score and Eco-Score: Consumers’ trade-offs when facing two sustainability labels. Food Quality and Preference. 2024;118:105200.

[22] Pink AE, Stylianou KS, Ling Lee L, Jolliet O, Cheon BK. The effects of presenting health and environmental impacts of food on consumption intentions. Food Quality and Preference. 2022;98:104501. 10.1016/j.foodqual.2021.104501 PMC13186242 42164907

[23] Howe HS, Fitzsimons GJ, Ubel P. Open Science Online Grocery: A Tool for Studying Choice Context and Food Choice. Journal of the Association for Consumer Research. 2022;7(4):393–402.

[24] Crosetto P, Lacroix A, Muller L, Ruffieux B. Nutritional and economic impact of five alternative front-of-pack nutritional labels: experimental evidence. European Review of Agricultural Economics. 2019;47(2):785–818.

[25] Davydenko M, Peetz J. Shopping less with shopping lists: Planning individual expenses ahead of time affects purchasing behavior when online grocery shopping. 2020;19(3):240–51.

[26] Pizzol M, Laurent A, Sala S, Weidema B, Verones F, Koffler C. Normalisation and weighting in life cycle assessment: quo vadis? The International Journal of Life Cycle Assessment. 2017;22(6):853–66.

