# Supplementary material for: Effects of environmental impact labels on the sustainability of food purchases: A randomised controlled trial in an experimental online supermarket
Source: PLoS One. 2024 Sep 3;19(9):e0309386. doi: 10.1371/journal.pone.0309386 (PMC11371233; doi:10.1371/journal.pone.0309386)
Supplement: S1 File — (DOCX) [file pone.0309386.s001.docx]

**Supplementary Materials: Effects of environmental impact labels on the sustainability of food purchases: A randomised controlled trial in an experimental online supermarket**

**Supplemental File 1. Categories of food groups displayed on the supermarket platform**

**Bakery**

Birthday & party cakes

Bread

Cakes & tarts

Croissants & breakfast bakery

Doughnut, cookies & muffins

Free from bread & cakes

From our in-store bakery

Naans & meal sides

Rolls & bagels

Scones, fruited & buns

Wraps, thins & pittas

**Dairy, eggs & chilled**

Cooked meats, olives & dips

Dairy & eggs

Deserts

Fresh soup

Pies, pasties & quiche

Pizza, pasta & garlic bread

Ready meals

Sandwiches & food to go

Savoury snacks

Vegetarian, vegan & dairy free

World foods, kosher & halal

**Drinks**

Milk & milk drinks

Chilled fruit juice & smoothies

Coffee

Fizzy drinks

Hot chocolate & milky drinks

Longer life juice & juice drinks

Squash & cordials

Tea

Water

**Food Cupboard**

Biscuits & crackers

Breakfast cereals

Confectionery

Canned, tinned & packaged foods

Cooking ingredients & oils

Cooking sauces & meal kits

Crisps, nuts & snacking fruit

Free from

Fruit & deserts

Jams, honey & spreads

Rice, pasta & noodles

Sugar & home baking

Table sauces, dressings & condiments

World foods

**Fruit & vegetables**

Fresh fruit

Fresh herbs & ingredients

Fresh salad

Fresh vegetables

Organic

Prepared fruit, veg & salad

**Frozen**

Chips, potatoes & rice

Desserts & pastry

Fish & seafood

Free from

Fruit, vegetables & herbs

Ice cream & ice

Meal & poultry

Pizza & garlic bread

Ready meals, pies & party food

Vegetarian

World foods

Yorkshire puddings & roast accompaniments

**Meat & fish**

Bacon & sausages

Beef

Chicken

Duck, game & venison

Fish & seafood

Ham & cooked meats

Lamb

Meat free

Pork & gammon Turkey

Yorkshire puddings & gravy

**Supplemental File 2.** Process of label development

- To develop the labels, we considered feedback from focus group sessions we ran, existing label examples promoting health or environmental sustainability, and key design components such as information, scales, colour and other visual cues. Due to resource constraints we were unable to systematically test each design component against each other so focused on three specific prototypes.
- We tested:
  - The A-E label, based on the Nutriscore example, which reflected feedback from focus groups that the label should be simple, familiar, and include multiple cues (i.e. A-E score, colours, and text stating this was an “environmental score”).
  - The petal label, conceived by one of us, Joseph Poore, and publicised in a 2018 [commentary](https://www.theguardian.com/environment/2018/oct/10/we-label-fridges-to-show-their-environmental-impact-why-not-food) in the Guardian (<https://www.theguardian.com/environment/2018/oct/10/we-label-fridges-to-show-their-environmental-impact-why-not-food>). This design explored whether, in contradiction to focus group feedback, a much more information-rich label would be capable of reducing the environmental impact score of participants’ shopping baskets.
  - The combined A-E + petal label, to explore whether giving study participants the option of a simple and more complicated design would assist or potentially confuse their ability to choose more environmentally sustainable foods.

Apart from the petal label, all of our ecolabels were designed by one or more of our co-authors using popular design software such as Adobe Photoshop.

**Supplemental File 3.** Woods experimental online supermarket platform welcome screen


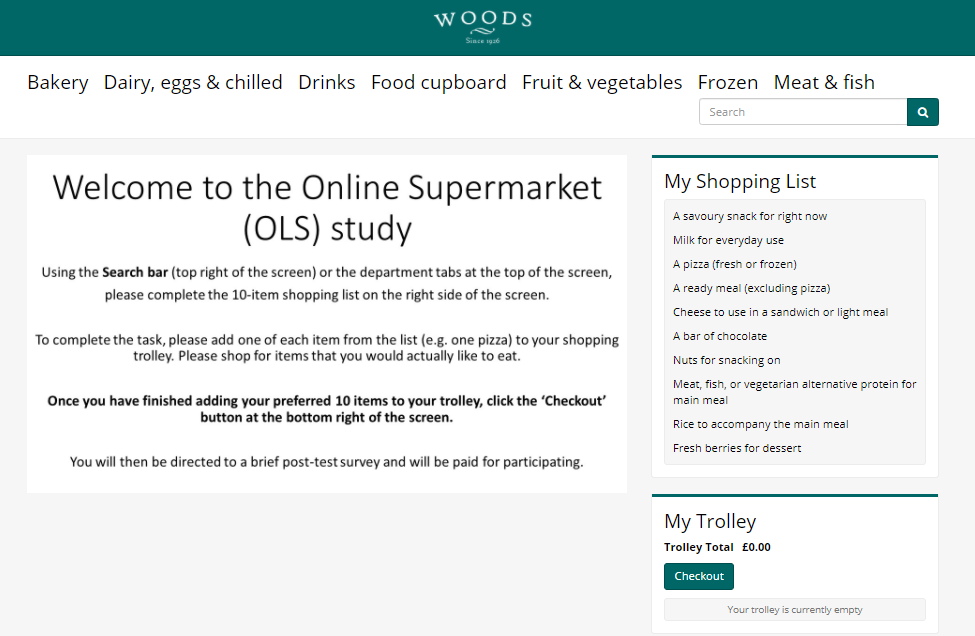


**Supplemental File 4. Post-test survey**

1. Demographic characteristics
   - Gender: o Male o Female o Other o Prefer not to say
   - Age (years): o Free text (range of 18-99) o Prefer not to say
2. Household size – “How many people live at your house, including you?
3. Regular shopping

“On average, how much do you spend on supermarket shopping per week?”

1. Online shopping experience

“How often, on average over the past year, have you shopped online for food or groceries to be delivered to you (e.g. Sainsburys.co.uk, Ocado.com, mysupermarket.co.uk)?”

- - Never or not in the last year
  - 1-3 times in the last year
  - 4-11 times in the last year
  - 1-3 per month
  - Once per week or more often.

1. “The online supermarket you have just used may have offered products which contained an environmental sustainability logo. Were you offered products which contained this logo during the shopping task today?”
   - “Yes/No”

5A. “Is this a feature you would like to have when you do your usual shopping?”

- - Strongly agree
  - Somewhat agree
  - Indifferent
  - Somewhat disagree
  - Strongly disagree

1. “How often do you look at the front of package nutrition labels when doing your usual grocery shop?”
   - Always
   - Often
   - Sometimes
   - Rarely
   - Never
2. “How often do you look at the ingredients list on food packaging when doing your usual grocery shop?”
   - Always
   - Often
   - Sometimes
   - Rarely
   - Never
3. “We are interested in your experience using the supermarket platform today. Please let us know if you had any difficulties locating the products on the shopping list or using the search functions.” [FREE TEXT ANSWER]
