# Supplementary material for: Effects of environmental impact labels on the sustainability of food purchases: A randomised controlled trial in an experimental online supermarket
Source: PLoS One. 2024 Sep 3;19(9):e0309386. doi: 10.1371/journal.pone.0309386 (PMC11371233; doi:10.1371/journal.pone.0309386)
Supplement: S2 File — (DOC) [file pone.0309386.s002.doc]

**CONSORT 2010 Flow Diagram**

Assessed for eligibility (N/A; ineligible ppts automatically withdrawn)

**Allocation**

**Analysis**

**Enrollment**

Allocated to control (n= 275)

♦ Received allocated intervention (n= 275)

Allocated to Combined labels (n= 271)

♦ Received allocated intervention (n= 271)

Analysed (n= 261)
♦ Excluded from analysis (incorrectly completed shopping task: n= 10)

Randomized (n= 1096)

Allocated to Petal labels (n= 273)

♦ Received allocated intervention (n= 273)

Allocated to A-E labels (n= 277)

♦ Received allocated intervention (n= 277)

Analysed (n= 265)
♦ Excluded from analysis (incorrectly completed shopping task: n= 12)

Analysed (n= 262)
♦ Excluded from analysis (incorrectly completed shopping task: n= 11)

Analysed (n= 263)
♦ Excluded from analysis (incorrectly completed shopping task: n= 12)
