# Supplementary material for: Effects of environmental impact labels on the sustainability of food purchases: A randomised controlled trial in an experimental online supermarket
Source: PLoS One. 2024 Sep 3;19(9):e0309386. doi: 10.1371/journal.pone.0309386 (PMC11371233; doi:10.1371/journal.pone.0309386)
Supplement: S1 Table — (PDF) [file pone.0309386.s003.pdf]

Study 1	1. Unadjusted Model			2. Adjusted model without interac			3. Model with only Gender interactions*				4. Model with only Age interactions			
**Variables**	R^2	Coef.	p-value	R2	Coef.	p-value	R2	Model fitting p-value by Likelihood Ratio Tests	Coef.	p-value	R2	Model fitting p-value by Likelihood Ratio Tests	Coef.	p-value
	0.0424			0.0546			0.0574	0.543			0.0655	0.2087		
Cohort = Petal		-3.9	**<0.001**		-4.1	**<0.001**			-4.6	**<0.001**			-10.5	**<0.001**
Cohort = A-E		-3.9	**<0.001**		-4.0	**<0.001**			-4.6	**<0.001**			-6.7	**0.017**
Cohort = Combined		-3.2	**<0.001**		-3.3	**<0.001**			-3.5	**<0.001**			-7.6	**0.006**
Gender = Male					0.0	0.966			-0.9	0.368			0.0	0.996
Gender = Other					-4.5	0.317			1.6	0.834			-4.4	0.331
Gender = Prefer not to say					3.4	0.657			3.8	0.620			4.1	0.601
Age = 21-40					3.4	**0.002**			3.2	**0.002**			0.0	0.985
Age = 41-60					2.7	**0.015**			2.6	**0.019**			-0.1	0.957
Age = 61+					3.7	**0.012**			3.5	**0.017**			-3.4	0.300
[Cohort = Petal] * [Gender = Male]									1.3	0.334			-	-
[Cohort = A-E] * [Gender = Male]									1.7	0.202			-	-
[Cohort = A-E] * [Gender = Other]									-9.0	0.342			-	-
[Cohort = Combined] * [Gender = Male]									0.5	0.718			-	-
[Cohort = Petal] * [Age = 21-40]													7.2	**0.017**
[Cohort = Petal] * [Age = 41-60]													5.9	0.057
[Cohort = Petal] * [Age = 61+]													10.2	**0.021**
[Cohort = A-E] * [Age = 21-40]													3.3	0.264
[Cohort = A-E] * [Age = 41-60]													2.1	0.485
[Cohort = A-E] * [Age = 61+]													6.0	0.167
[Cohort = Combined] * [Age = 21-40]													4.2	0.146
[Cohort = Combined] * [Age = 41-60]													4.3	0.156
[Cohort = Combined] * [Age = 61+]													11.4	**0.009**
Cohort ref = Control							*All other grp*gender interactions							
Age ref = 18-20	All labels sig. different			Age a signficant factor			empty or omitted				No age*label interactions			
Gender ref = Female	from control			No gender effect										
							No label*gender interaction							
