# Supplementary material for: Effects of environmental impact labels on the sustainability of food purchases: A randomised controlled trial in an experimental online supermarket
Source: PLoS One. 2024 Sep 3;19(9):e0309386. doi: 10.1371/journal.pone.0309386 (PMC11371233; doi:10.1371/journal.pone.0309386)
Supplement: S2 Table — (DOCX) [file pone.0309386.s004.docx]

**Supplemental Table 2a.** Comparison of the mean environmental impact score between trial groups.

| **Control** | **Petal vs Control** | **A-E vs Control** | **Combined vs Control** |
| --- | --- | --- | --- |
| **Mean (SD)** | **Mean difference**  **(95% CI)** | **Mean difference**  **(95% CI)** | **Mean difference**  **(95% CI)** |
| 61.9 + 5.95 | -3.94 (-5.25, -2.62)** | -3.85 (-5.16, -2.54)** | -3.19 (-4.51, -1.87)** |
| *Note.* Values are arithmetic means + SDs in column 1 and mean differences (95% CIs) in columns 2, 3, and 4. **p* <0.01, **p <=0.001 | | | |

**Supplemental Table 2b.** Exploratory analyses (Mean environmental impact score for participants who bought 10 items (n=930); and total environmental impact scores for participants buying 5 or more items from the shopping list (n=1051))

|  | **Control** | **Petal vs Control** | **A-E vs Control** | **Combined vs Control** |
| --- | --- | --- | --- | --- |
|  | **Mean (SD)** | **Mean difference**  **(95% CI)** | **Mean difference**  **(95% CI)** | **Mean difference**  **(95% CI)** |
| **10 items** | 62.3 (5.4) | -3.84  (-5.20, -2.47)** | -4.23  (-5.60, -2.86)** | -3.56  (-4.93, -2.19)** |
| **Total environmental impact score** | 70.7 (27.1) | -9.32  (-14.22, -4.42)** | -12.36  (-17.24, -7.47)** | -10.33  (-15.23, -5.42)** |

*Note.* Values are arithmetic means + SDs in column 1 and mean differences (95% CIs) in columns 2, 3, 4, and 5. **p* <0.01, **p <=0.001
