# Supplementary material for: Effects of environmental impact labels on the sustainability of food purchases: A randomised controlled trial in an experimental online supermarket
Source: PLoS One. 2024 Sep 3;19(9):e0309386. doi: 10.1371/journal.pone.0309386 (PMC11371233; doi:10.1371/journal.pone.0309386)
Supplement: S3 Table — (DOCX) [file pone.0309386.s005.docx]

**Supplementary Table 3.** Percentage (n) of purchased items falling into each environmental impact grade, and the mean (s.d.) score on each environmental indicator of purchased items by grade.

|  | | Control | Petal | A-E | Combined | **Total/ Mean** |
| --- | --- | --- | --- | --- | --- | --- |
| A | n (%) | 391 (15.39) | 596 (23.70) | 585 (22.63) | 567 (22.51) | **2139 (21.06)** |
|  | GHGs | 0.21 (0.33) | 0.19 (0.30) | 0.22 (0.41) | 0.22 (0.39) | **0.21**  **(0.36)** |
|  | Biodiversity | 9.09 (24.77) | 6.36 (20.56) | 7.30 (21.1) | 6.74 (19.69) | **7.22 (21.32)** |
|  | Eutrophication | 0.70 (1.11) | 0.59 (1.01) | 0.78 (1.38) | 0.85 (1.46) | **0.76 (1.26)** |
|  | Water use | 431.11 (622.91) | 455.16 (722.75) | 425.08 (679.45) | 459.62 (701.32) | **443.72 (687.53)** |
| B | n (%) | 488  (19.21) | 566 (22.50) | 585 (22.63) | 581 (23.06) | **2220 (21.85)** |
|  | GHGs | 0.41 (0.70) | 0.38 (0.64) | 0.36 (0.61) | 0.33 (0.48) | **0.37 (0.61)** |
|  | Biodiversity | 8.3 (17.70) | 8.48 (15.33) | 7.81 (16.53) | 7.79 (18.73) | **8.08 (17.10)** |
|  | Eutrophication | 1.59 (2.40) | 1.52 (2.24) | 1.47 (2.16) | 1.32 (1.76) | **1.47 (2.15)** |
|  | Water use | 882.03 (819.21) | 867.56 (783.45) | 919.34 (779.29) | 904.75 (816.09) | **894.12 (798.68)** |
| C | n (%) | 486 (19.13) | 401 (15.94) | 434 (16.79) | 432 (17.15) | **1753 (17.26)** |
|  | GHGs | 0.46 (0.64) | 0.41 (0.48) | 0.42 (0.52) | 0.43 (0.53) | **0.43 (0.55)** |
|  | Biodiversity | 12.08 (23.53) | 12.46 (25.43) | 10.29 (18.57) | 9.39 (17.52) | **11.06 (21.53)** |
|  | Eutrophication | 1.98 (2.72) | 1.70 (2.12) | 1.81 (2.19) | 1.92 (2.62) | **1.86 (2.44)** |
|  | Water use | 1560.23 (1475.99) | 1471.98 (1313.92) | 1490.28 (1342.53) | 1621.37 (1374.13) | **1537.79 (1382.25)** |
| D | n (%) | 684 (26.93) | 605 (24.06) | 617 (23.87) | 518 (20.56) | **2424 (23.86)** |
|  | GHGs | 0.55 (0.61) | 0.54 (0.67) | 0.52 (0.55) | 0.56 (0.66) | **0.54 (0.62)** |
|  | Biodiversity | 14.13 (28.74) | 12.93 (23.19) | 12.36 (24.16) | 14.89 (30.10) | **13.54 (26.64)** |
|  | Eutrophication | 2.25 (2.38) | 2.31 (3.06) | 2.19 (2.53) | 2.48 (3.13) | **2.29 (2.77)** |
|  | Water use | 2264.02 (2867.14) | 2199.97 (2773.62) | 1928.20 (2001.00) | 2159.75 (2493.92) | **2140.27 (2568.40)** |
| E | n (%) | 491 (19.33) | 347 (13.80) | 364 (14.08) | 421 (16.71) | **1623 (15.98)** |
|  | GHGs | 0.83 (0.87) | 0.83 (0.87) | 0.87 (0.86) | 0.78 (0.84) | **0.82 (0.86)** |
|  | Biodiversity | 20.12 (26.21) | 21.81 (32.15) | 22.75 (30.96) | 22.32 (32.57) | **21.64 (30.30)** |
|  | Eutrophication | 3.50 (3.53) | 3.59 (3.70) | 3.86 (4.08) | 3.43 (3.78) | **3.58 (2.69)** |
|  | Water use | 5531.07 (6998.52) | 5158.03 (6889.81) | 4854.35 (5997.28) | 5069.00 (7183.97) | **5179.68 (6811.76)** |
| **Total/ Mean** | **n (%)** | **2540 (100)** | **2515 (100)** | **2585 (100)** | **2519 (100)** | **10159 (100)** |
|  | **GHGs** | **0.51 (0.69)** | **0.44 (0.63)** | **0.45 (0.62)** | **0.45 (0.62)** | **0.46 (0.64)** |
|  | **Biodiversity** | **13.00 (25.13)** | **11.52 (23.47)** | **11.30 (22.78)** | **11.72 (24.79)** | **11.88 (24.06)** |
|  | **Eutrophication** | **2.08 (2.72)** | **1.83 (2.65)** | **1.88 (2.65)** | **1.91 (2.75)** | **1.92 (2.69)** |
|  | **Water use** | **2213.23 (3912.68)** | **1778.68 (3339.74)** | **1698.24 (2911.74)** | **1881.50 (3588.07)** | **1892.36 (3460.23)** |
